# Superior room-temperature ductility of typically brittle quasicrystals at small sizes

**DOI:** 10.1038/ncomms12261

**Published:** 2016-08-12

**Authors:** Yu Zou, Pawel Kuczera, Alla Sologubenko, Takashi Sumigawa, Takayuki Kitamura, Walter Steurer, Ralph Spolenak

**Affiliations:** 1 Department of Materials, Laboratory for Nanometallurgy, ETH Zurich, Vladimir-Prelog-Weg 5, CH-8093 Zurich, Switzerland; 2 Department of Materials, Laboratory of Crystallography, ETH Zurich, Vladimir-Prelog-Weg 5, CH-8093 Zurich, Switzerland; 3 Department of Mechanical Engineering and Science, Graduate School of Engineering, Kyoto University, Nishikyo-ku, Kyoto 615-8540, Japan

## Abstract

The discovery of quasicrystals three decades ago unveiled a class of matter that exhibits long-range order but lacks translational periodicity. Owing to their unique structures, quasicrystals possess many unusual properties. However, a well-known bottleneck that impedes their widespread application is their intrinsic brittleness: plastic deformation has been found to only be possible at high temperatures or under hydrostatic pressures, and their deformation mechanism at low temperatures is still unclear. Here, we report that typically brittle quasicrystals can exhibit remarkable ductility of over 50% strains and high strengths of ∼4.5 GPa at room temperature and sub-micrometer scales. In contrast to the generally accepted dominant deformation mechanism in quasicrystals—dislocation climb, our observation suggests that dislocation glide may govern plasticity under high-stress and low-temperature conditions. The ability to plastically deform quasicrystals at room temperature should lead to an improved understanding of their deformation mechanism and application in small-scale devices.

## Abstract (web summary)

Quasicrystals are typically stiff and hard, but also brittle in bulk form at room temperature. Here, authors observe ductile behaviour in quasicrystalline pillars of submicron diameter and provide evidence for dislocation-based plasticity at intermediate length scales and room temperature as an explanation.

## Graphical abstract

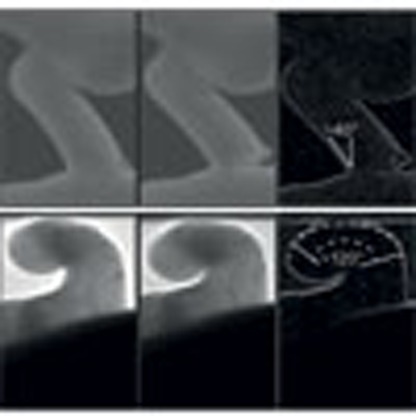

In materials science, plasticity describes the non-reversible deformation of a solid in response to applied forces and determines the ability of a material to change its shape permanently without breaking. Regular crystalline materials, including most metals and ceramics, are generally plastically deformed through dislocation motion^1^ or twinning^2^. The plasticity of amorphous solids, such as metallic glasses, is based on the formation and propagation of shear bands^3^. In quasicrystals^4^, despite their lack of periodicity, plastic deformation can also be achieved by dislocation activities^5^. In contrast to the situation in periodic crystals, every movement of a dislocation in a quasicrystal creates a cloud behind, which is called phason fault^6^. As a consequence, the dislocation motion gets hindered and the material appears brittle. Although a great variety of quasicrystals have been synthesized^7^,^8^, and some have even been discovered in nature^9^, and found to be technologically interesting^10^,^11^,^12^,^13^ and useful^14^, only few of them can be found in applications so far, mainly limited by their poor ductility and formability at room temperature. Hence, improving the room-temperature ductility of quasicrystals is not only of academic interest but also essential for technological applications.

Early studies of the plastic deformation of quasicrystals focused on an easily grown icosahedral quasicrystal, i-Al–Pd–Mn, in the high-temperature regime above ∼600 °C (∼70% of its melting temperature). These studies demonstrated that the plastic deformation of i-Al–Pd–Mn was dominated by dislocation climb—with the Burgers vector out of the plane of dislocation motion, rather than dislocation glide—with the Burgers vector restricted in the plane of dislocation motion^15^. It is generally believed that dislocation climb is a much easier deformation mode in quasicrystals than dislocation glide^16^. Although there are some hints that the glide motion may be possible in low-temperature conditions as suggested by numerical simulations^17^ or under high hydrostatic pressures^18^, the required stress to activate glide is extremely high, on the order of 1/10 of its shear modulus—a stress level generally leading to fracture without showing any ductility. It has been a long-standing question concerning the deformation mechanism in quasicrystals at room temperature. Despite several investigators have sought to explore the plastic deformation of quasicrystals at or near room temperature using indentation or by confining gas or solid pressures^19^,^20^,^21^,^22^, so far there has been no common conclusion: the explanations include shear banding similar to metallic glasses^23^, phase transformation^24^,^25^, grain-boundary glide^21^, pure dislocation climb^22^, dislocation climb dominant^26^ and crystallization^27^. Therefore, one has to conclude that the plastic deformation of quasicrystals under a wide range of temperatures and pressures has been poorly understood—much in contrast to crystalline and amorphous solids. Two fundamental questions are still open: can steady-state plastic deformation be achieved at room temperature? If so, what is the underlying deformation mechanism?

Unveiling room-temperature plasticity in quasicrystals hence relies on a new method to suppress fracture before plastic yielding in a simple loading experiment. Our strategy is to increase the fracture strength over the yield strength in a quasicrystal by reducing the sample size. Although similar methods have been explored for other brittle materials such as ceramics^28^ and metallic glasses^29^,^30^, it has, to our knowledge, not previously been reported for quasicrystals—a large family of unusual solids. In this study, we demonstrate a brittle-to-ductile transition in quasicrystals at room temperature due to a sample size reduction—a submicron-sized quasicrystal pillar exhibits superior ductility at room temperature. Furthermore, we suggest that dislocation glide may control the plastic deformation of quasicrystals at room temperature and attempt to shed light on the underlying deformation mechanism in the low-temperature regime.

## Results

### A model to predict brittle-to-ductile transition

**Figure 1:**
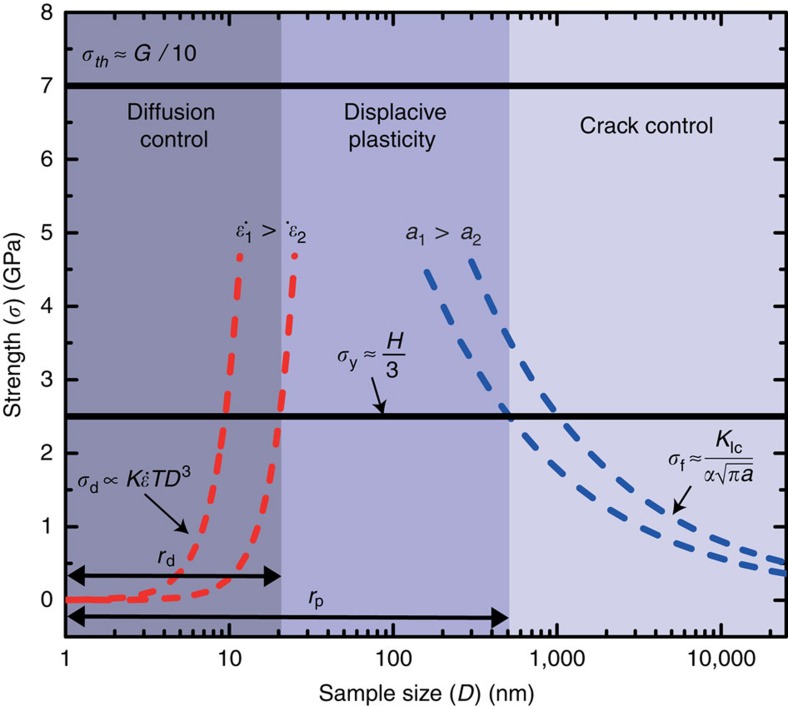
Deformation map for small-scale i-Al–Pd–Mn quasicrystals. Semi-quantitative predictions for room temperature deformation. If *D*>*r*_p_, defined as the intersection of the fracture strength, *σ*_f_ (the blue dashed lines), and the yield strength, *σ*_y_ (the black solid line), the material fails by cracking without notable plasticity, following the Griffith's criterion^37^, *σ*_f_=*K*_Ic_/[*α*(*πa*)^−1/2^] with *K*_Ic_ the fracture toughness of the material, *α* a geometrical parameter on the order of unity and *a* the size of pre-existing cracks or flaws. The *σ*_f_ shows a smaller-is-stronger trend. If *D*<*r*_d_, defined as the intersection of *σ*_y_ and *σ*_d_, the diffusion governs the strength, following 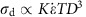 with *K*, surface diffusivity, 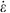, strain rate, and *T*, temperature. The *σ*_d_ shows a smaller-is-weaker phenomenon. In between *r*_p_ and *r*_d_, the curves of *σ*_f_, *σ*_d_ and *σ*_y_ are crossed and define a zone controlled by displacive deformation. The size range of this zone may vary by flaw sizes and strain rates, as illustrated.

To estimate at what size range a typically brittle quasicrystal may become ductile, we compared the different deformation mechanisms as a function of the sample size: dislocation activities, crack propagation^31^ and mass transport by diffusion^32^. We identified three deformation regimes: cracking-controlled, displacive-deformation-controlled (dislocations or shear bands) and diffusion-controlled, as illustrated in Fig. 1. We estimated the critical size, *r*_p_, for the brittle-to-ductile transition to be ∼500 nm, and the size of the diffusion-controlled zone, *r*_d_, to be around 10 nm (see the detailed analysis in Methods section at the end of the article). Our targeted sample size to attain steady-state plasticity thus falls in a range from ∼100 to ∼500 nm.

### Micro-compression of small-sized quasicrystal pillars

**Figure 2:**
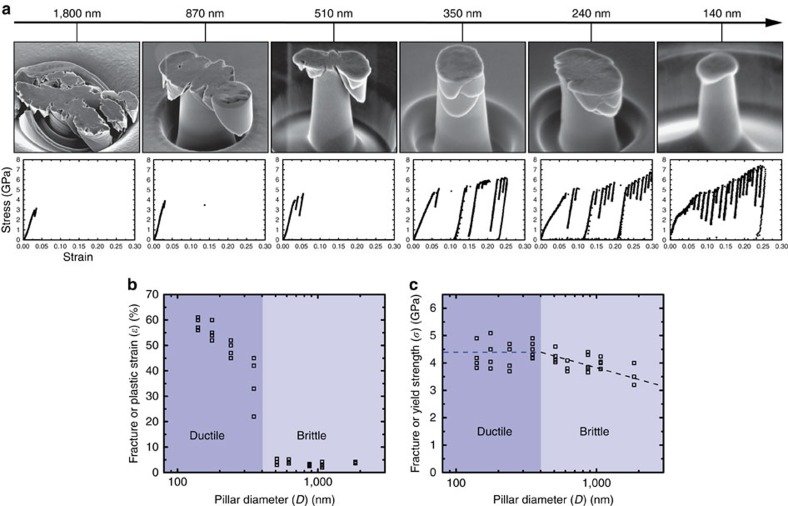
Micro-compression of single-quasicrystalline i-Al–Pd–Mn pillars. Pillar diameters range from ∼2 μm to ∼150 nm. (**a**) Typical SEM images of the post-deformed pillars, showing a brittle-to-ductile transition with the critical size between 350 and 510 nm. The corresponding engineering stress–strain curves are presented below. (**b**) The fracture strain or plastic strain as a function of the pillar diameter, indicating a brittle-to-ductile transition. (**c**) The fracture strength or yield strength as a function of the pillar diameter. In the brittle regime, the strength increases slightly with decreasing the pillar diameter; in the ductile regime, the strength is almost independent of the sample size.

In our experiments, we compressed single-quasicrystalline i-Al–Pd–Mn pillars with diameters ranging from ∼1.8 μm to ∼150 nm. We observed a brittle-to-ductile transition with the critical pillar diameter between 510 and 350 nm (Fig. 2a): the 1.8-μm pillar exhibits a catastrophic failure at ∼3% compressive strain; the 870- and 510-nm pillars show cracks at about 45° along the loading direction, failing at ∼6% strain; when the pillar diameter is below 500 nm, the pillars present significantly improved ductility with compressive strains over 50% and without any cracking. The 400- and 200-nm pillars clearly show deformation bands, while the 140-nm pillar reveals the deformation localized at the upper part of the pillar. All the corresponding stress–strain curves exhibit a displacement-burst phenomenon (Fig. 2a), which is generally observed in metals^33^,^34^ and metallic glasses^29^,^30^. The 140- and 240-nm pillars exhibit earlier plastic yielding than the other pillars, which could be due to localized deformation on the pillar top region or the lateral friction between the indenter tip and the top surface of the pillar. Here, the flow stresses after the first displacement bursts were used to give a best estimation of their yield strengths. How the fracture strain or maximum plastic strain changes by decreasing the sample size also demonstrates the brittle-to-ductile transition between 510 and 350 nm (Fig. 2b). When the pillar diameter is smaller than 350 nm, no cracking is observed in our experiments. Regarding the size dependence of strength, the fracture strength increases from ∼3.5 to ∼4.5 GPa with decreasing pillar diameters in the brittle regime, while the yield strength (the flow stress at the first displacement burst) is about 4.5 GPa in the ductile regime (Fig. 2c).

### *In situ* bending tests of small-sized quasicrystal pillars

**Figure 3:**
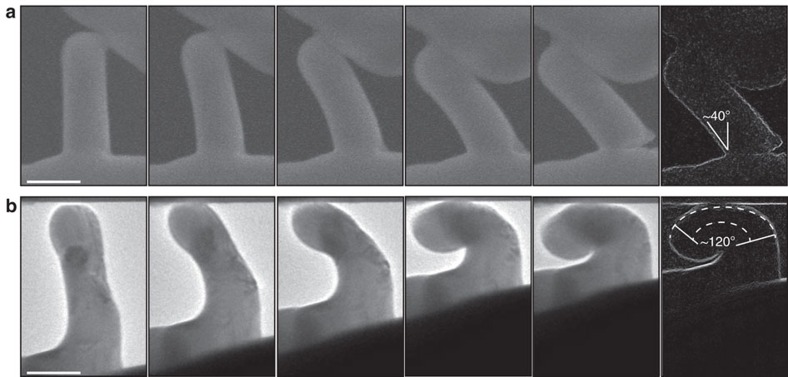
*In situ* SEM and TEM of i-Al–Pd–Mn pillars during bending tests. (**a**) SEM snapshots captured during the bending test of a pillar with the diameter of ∼300 nm. An initial crack occurs near the pillar base at the bending angle of ∼20–30° and eventual fracture happens at the bending angle of ∼40°. (**b**) TEM snapshots during bending tests on a pillar in the diameter of ∼100 nm, showing a homogenous deformation without any fracture, and the maximum tensile strain at the pillar centre estimated to be over 50%. Scale bars, 300 nm (**a**) and 100 nm (**b**).

Brittle materials usually show higher ductility in compression than tension. To examine the tensile ductility of the quasicrystal pillars but avoid the complex experimental setup of the tensile test for sub-micrometer-sized samples, we employed micro-bending tests to induce an asymmetrical stress distribution and compare the bending ductility of the pillars in different sizes. The *in situ* scanning electron microscopy (SEM) bending of a 300-nm pillar shows that the deformation localizes near the pillar base by necking. We detected that the crack forms at the bending angle of ∼20–30°, and eventually fails in a catastrophic feature at the bending angle of ∼40° (Fig. 3a). The *in situ* transmission electron microscopy (TEM) bending of a 100-nm pillar shows in a rather homogenous deformation without any cracking and fracture (Fig. 3b). The longitudinal tensile strain near the pillar centre is estimated to be over 50%. The strain bands' motion during the tests implies dislocation activity during the deformation (Supplementary Fig. 1).

### TEM characterization and diffraction simulations

**Figure 4:**
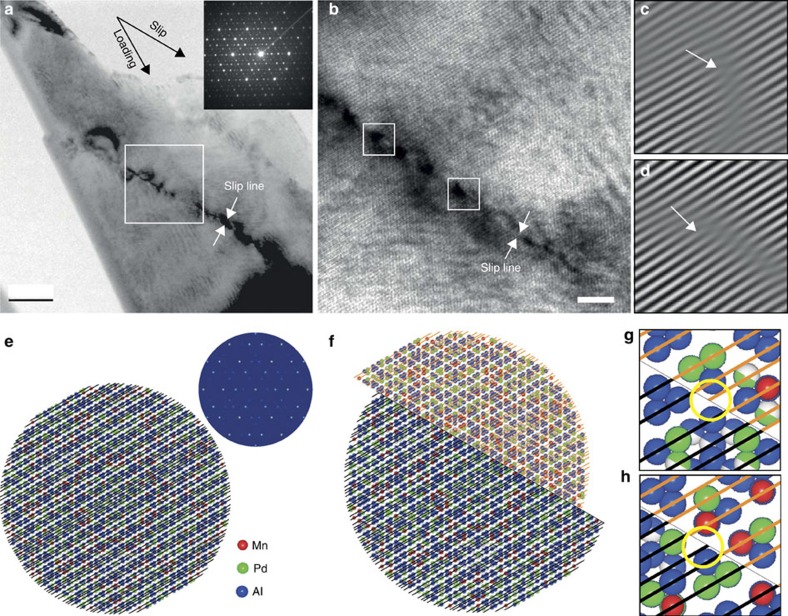
Locally deformed region in i-Al–Pd–Mn pillar. TEM images observed along a threefold axis. (**a**) A typical bright-field TEM image showing a narrow and straight band traversing the pillar and the corresponding electron diffraction pattern. The loading direction is along a twofold axis and the slip direction is along another twofold axis. (**b**) The high-resolution TEM image shows the deformation band with a thickness of ∼2–5 nm and strain contrast modulations along the line (periodic dark regions along the band). The rest of the area is nearly defect-free. (**c**,**d**) The inverse Fourier transformation of the regions marked in **b**, emphasizing the very localized and periodic lattice distortions along the deformation band. The inserted fringes are indicated by arrows. (**e**) The atomic model of i-Al–Pd–Mn projected along the threefold axis with its calculated diffraction pattern to be compared with the experimental one in **a**, before the deformation. (**f**) A schematic view in projection of the model after shear deformation, with the same loading and slip directions as shown in **b**. (**g**,**h**) The local mismatches between the quasi-lattice planes, where the strain is concentrated, and correlating (**c**) and (**d**) (the black and orange lines indicate quasi-lattice planes and the circles indicate dislocations). Scale bars, 50 nm (**a**) and 10 nm (**b**).

A representative bright-field TEM image reveals the upper part of a deformed pillar along a threefold axis (Fig. 4a). We find a slip line through the pillar and a step at the pillar edge. The loading direction is along a twofold axis and the slip plane contains another twofold axis. The high-resolution TEM image shows a very narrow band of ∼2–5 nm in thickness. Along the band, there are strain-contrast modulations with a nearly equal distance of ∼2–5 nm and the area surrounding deformation band is nearly defect-free. (Fig. 4b; Supplementary Fig. 2). We do not observe any evidence of melting, crystallization, phase transformation or cracking that was used to explain room-temperature deformation in quasicrystals. Different from the deformation bands formed in i-Al–Pd–Mn under hydrostatic pressures and at room temperature^18^, the bands observed here are much narrower and contain a much lower defect density. Using the inverse Fourier transformed images (Fig. 4c,d from the boxed areas in Fig. 4b), we identify a few inserted fringes along the deformation band. These inserted fringes indicate the distortions caused by dislocations. Together with the sharp deformation band and the step at the pillar edge (Fig. 4a), the fringes suggest that their Burgers vectors may contain the components along the slip or shear direction—dislocation glide might have occurred. Our atomic model of i-Al–Pd–Mn quasicrystal matches the orientation of the sample before and after deformation, respectively (Fig. 4e,f). Along the slip line shown in Fig. 4f, we can identify the mismatch region generated by the dislocation glide due to the local shear between the quasi-lattice planes (Fig. 4g,h). Such discontinuous quasi-lattice planes could be interpreted as dislocations with Burgers vector along the slip direction, which compares to the fringe patterns in Fig. 4c,d. The strain contrast shown in Fig. 4b could be attributed to strain fields of the dislocations or related phason faults left behind. This is a strong indication that the plasticity of quasicrystals at room temperature can be dominated by dislocation glide.

## Discussion

The results shown in Figs 2 and 3 confirm that i-Al–Pd–Mn pillars are capable of both excellent ductility (compressive and tensile) and maintaining high strength when the pillar diameter is below about 500 nm. To our knowledge, this result has never been reported for quasicrystals before. The quasicrystal fine-scale pillars exhibit minor size dependence of strength and a deformation morphology with wavy features (see high-resolution SEM images in Supplementary Fig. 3), which is more similar to metallic glasses^29^,^30^ than to metals^35^. Nevertheless, we show that the quasicrystal plasticity at room temperature is still controlled by dislocation mechanisms.

Although in quasicrystals climb leads to the removal or insertion of so-called ‘worms' without overlaps or open spaces^15^, this process requires thermal activation. At room temperature, the atomic diffusion in quasicrystals is generally believed to be inhibited. Dislocation glide, however, may be active and even dominate under high-stress and low-temperature conditions, generating a high density of heavily distorted zones in the wake of the dislocation glide. The approach of reducing sample size to enhance the ductility of otherwise brittle quasicrystals may pave way to fundamentally understand the deformation mechanism of quasicrystals at room temperature, possibly at even lower temperatures and for all the other types of quasicrystals^36^.

**Figure 5:**
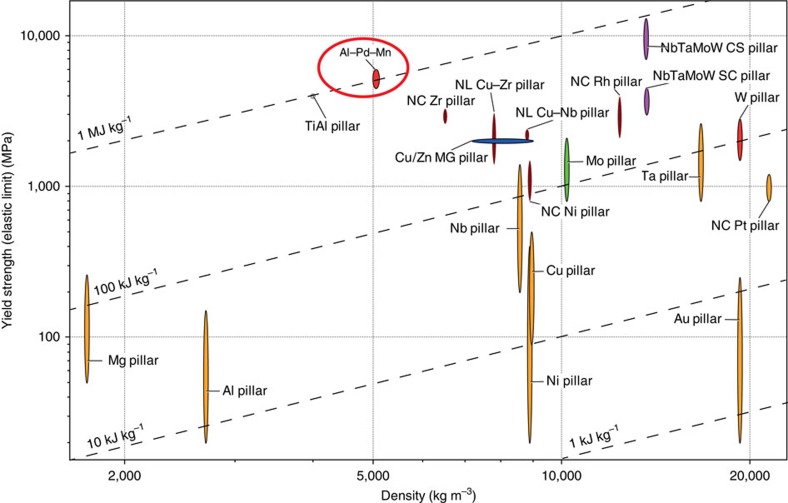
Strength comparison with other metallic and metallic-glass pillars. Ashby map (designed with CES EduPack 2014) of yield strength versus density, indicating that i-Al–Pd–Mn quasicrystal pillars exhibit, to our knowledge, the highest specific strength or the strength-to-density ratio (CS, single crystalline; NC, nanocrystalline; NL, nanolamellar; MG, metallic glass). The strength levels of i-Al–Pd–Mn quasicrystal pillars are from Fig. 2c. Literature data for pillar strengths: pure metals Au^34^,^44^, Al^45^, Ni^46^, Cu^47^, Nb, Ta, Mo and W^48^,^49^ and Mg^50^, TiAl^51^, nanocrystalline (nc) Cu^52^, Ni^53^, Ni–W^54^, Pt^55^ and Rh^56^ pillars, NbTaMoW high-entropy alloys^57^,^58^, and metallic glasses (for example, Cu- and Zn-based ones^59^).

Towards technological applications, fine-scale quasicrystals are attractive not only due to combining high strength with ductility but also because they offer extraordinary specific strength (strength divided by density or elastic energy density, ∼1 MJ kg^−1^) among metallic micro/nano-pillars reported to date (Fig. 5), which might be used to store elastic energy. Small dimensional quasicrystals having superior strength and ductility, together with their interesting functional properties, may also enable components that are both structurally and functionally useful in micro- or nano-electromechanical systems. While much work remains to optimize their properties, our observation of superior room-temperature ductility in quasicrystals motivates further fundamental and technological exploration.

## Methods

### Sample preparation and characterization

An initial compact of composition Al_70_Pd_21.5_Mn_8.5_ was prepared from pure metals (Al 99.9999%, Pd 99.9%, Mn 99.95%). The sample was pre-alloyed in an arc furnace, and subsequently placed in an Al_2_O_3_ crucible and sealed in a quartz glass ampoule under an Ar atmosphere. The heat treatment consisted of the following steps: heating to 1323 K (above its melting temperature), slow cooling to 1083 K at the rate of 30 K h^−1^, annealing at 1,083 K for 150 h, and subsequent quenching in water. The composition of the resulting sample was confirmed using energy dispersive X-ray spectroscopy. The X-ray powder diffraction pattern (Supplementary Fig. 4) and TEM diffraction patterns (Supplementary Fig. 5) indicate that the resulting sample is a single-phase icosahedral quasicrystal, which is comparable to that in literature^60^.

The prepared i-Al–Pd–Mn was thermodynamically stable with an average grain size of about 300 μm and was also highly isotropic. We fabricated single-quasicrystalline pillars, in cylindrical shapes, from a coarse grain in a well-polished i-Al–Pd–Mn sample using a FIB system (Helios Nanolab 600i, FEI): a coarse milling condition of 30 kV and 80 pA and a final milling condition of 5 kV and 7 pA. The diameters of the FIB-milled pillars are in the range of ∼150 nm to ∼2 μm and the aspect ratios are ∼3.0–4.5. A taper of 2–3° was generally observed and the top diameter of the pillar was chosen to calculate stress.

### Micro-mechanical testing

We used the nanoindenter (Hysitron Inc., USA) with a diamond flat-punch tip (5 μm in diameter, Synton-MDP, Switzerland) to compress the pillars in a displacement control mode and the strain rate of 2 × 10^−3 ^s^−1^ by feedback mechanism. At least four pillars for each size were compressed. The deformed pillars were imaged using a high-resolution SEM (Magellan, FEI). For the post-mortem TEM characterization, the deformed pillars were thinned down to a lamella by ion milling, lift-out, thinning and polishing in the FIB system. Their cross-sections were then examined using a TEM (Tecnai F30, FEI, operated at 300 kV). *In situ* SEM and TEM bending tests were carried out using a nano-manipulator (Kleindiek, Germany) fitted to a SEM (Hitachi SU 8200) and an indenter holder (Nanofactory Instruments AB, SA2000N) fitted to a TEM (JEOL JEM-2100), respectively, with a displacement rate of ∼5 nm s^−1^.

### Prediction of the brittle-to-ductile transition

In a brittle material, the fracture strength, *σ*_f_, follows the Griffith's criterion^37^, as *σ*_f_=*K*_Ic_/[*α*(*πa*)^1/2^] with *K*_Ic_ the fracture toughness of the material, *α* a geometrical parameter on the order of unit and *a* the size of pre-existing cracks or flaws. Statistically, larger samples are more likely to contain larger flaws, or weaker links, and consequently, smaller samples usually exhibit higher fracture strengths than the large ones—the size effect due to the Weibull statistics^38^. Because the fracture strength, *σ*_f_, cannot rise above the yield strength, *σ*_y_, below a certain length scale plastic flow may determine the strength. The intersection between the curves for *σ*_f_ and *σ*_y_ provides a critical size, *r*_p_, for a brittle-to-ductile transition, as illustrated in Fig. 1a. Assuming that the largest pre-existing cracks or flaws is one order of magnitude smaller than the sample dimension, we can obtain *r*_p_ of ∼500 nm for i-Al–Pd–Mn, using *α*, ∼1, *K*_Ic_, ∼1.25 MPa × *m*^1/2^ (ref. 39) and the hardness, *H*, ∼8.5 GPa (ref. 39). However, further reduction of the sample size down to the nanometre scale leads to a significant increase of the surface-to-volume ratio, and surface diffusion may control the plastic flow, resulting in a reduced strength. In a relation similar to the Coble creep^40^, the diffusion strength, *σ*_d_, reflects a ‘smaller-is-weaker' phenomenon. The crossover between *σ*_d_ and *σ*_y_ defines a diffusion-controlled zone with the length scale of *r*_d_ (Fig. 1a). Although it is difficult to calculate the exact value of *r*_d_ due to the lack of available literature data, recent studies on Al_90_Fe_5_Ce_5_ metallic glass^41^ and pure Sn^42^ demonstrate that diffusion controls plasticity below the sample sizes of 20 nm and 130 nm, respectively, at a strain rate of ∼10^−3^ s^−1^ and room temperature. Hence, we estimate the *r*_d_ for i-Al–Pd–Mn as a few tens of nanometres (definitely smaller than 100 nm), under similar experimental conditions. On the basis of this analysis, our targeted sample size to attain steady-state plasticity falls in a range from ∼100 to ∼500 nm.

### Diffraction simulations

We used the Quiquandon–Gratias atomic model (∼70 Å in diameter) of icosahedral Al–Pd–Mn^43^. We oriented the model along the threefold axis (high-magnification image in Supplementary Fig. 6), and calculated the diffraction pattern and compared with the experimental electron diffraction pattern. This agreement indicates that the orientation of our model matches the orientation of the sample.

### Data availability

The data that support the findings of this study are available from the corresponding author upon request.

## Additional information

**How to cite this article**: Zou, Y. *et al.* Superior room-temperature ductility of typically brittle quasicrystals at small sizes. *Nat. Commun.* 7:12261 doi: 10.1038/ncomms12261 (2016).

## Supplementary Material

Supplementary Information Supplementary Figures 1-6
