# Supplementary material for: Superior room-temperature ductility of typically brittle quasicrystals at small sizes
Source: Nat Commun. 2016 Aug 12;7:12261. doi: 10.1038/ncomms12261 (PMC4990631; doi:10.1038/ncomms12261)
Supplement: Supplementary Information — Supplementary Figures 1-6 [file ncomms12261-s1.pdf]

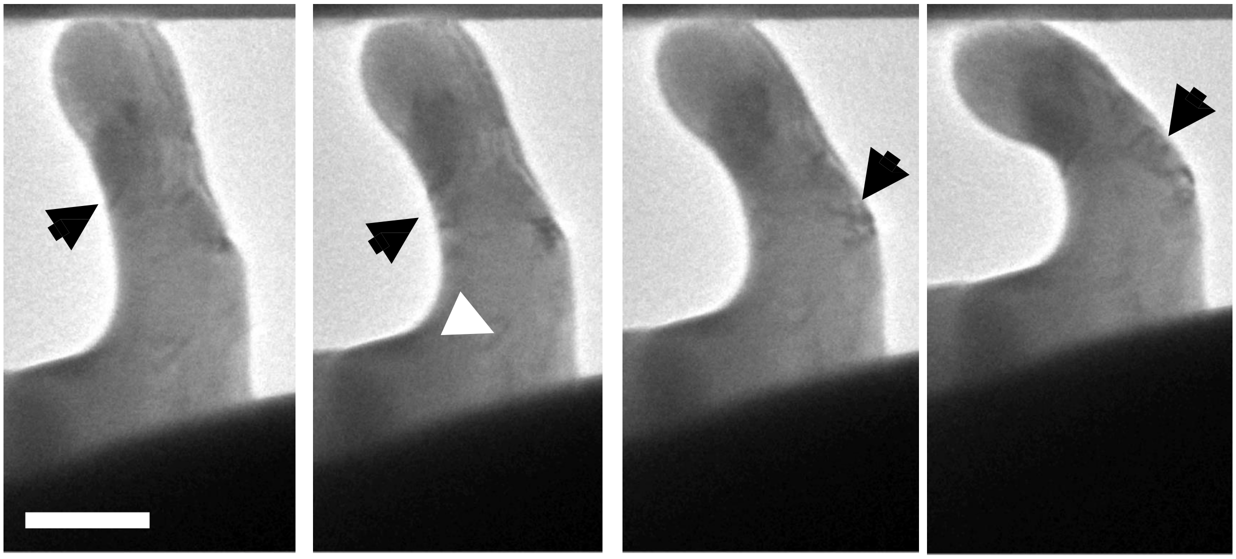

Supplementary Figure 1. Progressive TEM snapshots during bending test. The arrows indicate regions of dislocation-like contrast. Scale bar, 100 nm.

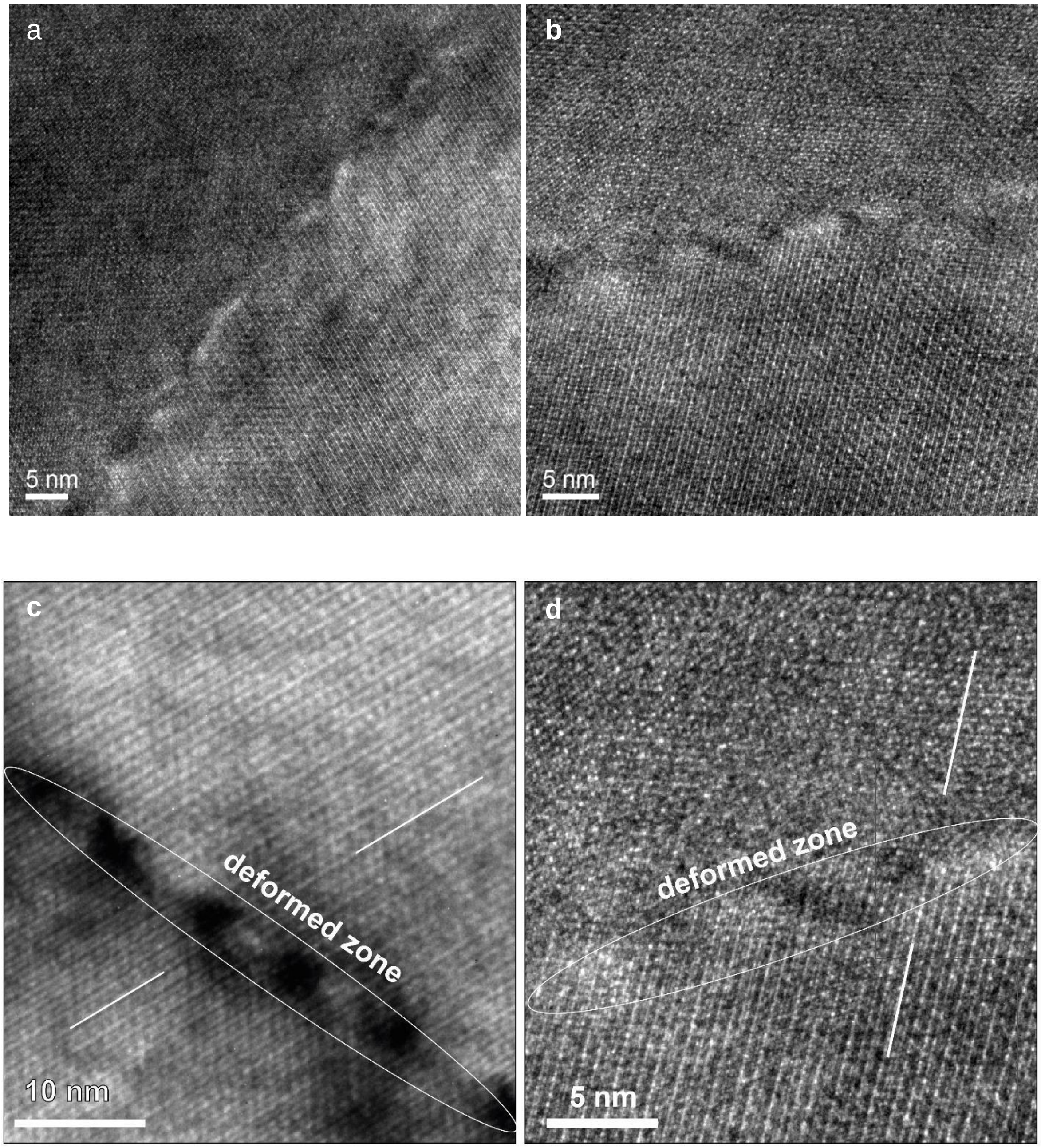

Supplementary Figure 2. High-resolution TEM images of deformation band. Band has thickness of ~2-5 nm and strain concentrations lie along the indicated lines. The surrounding area is nearly defect free. The areas above and below the deformation zone are coherent. Scale bars, a, 10 nm, and the others, 5 nm.

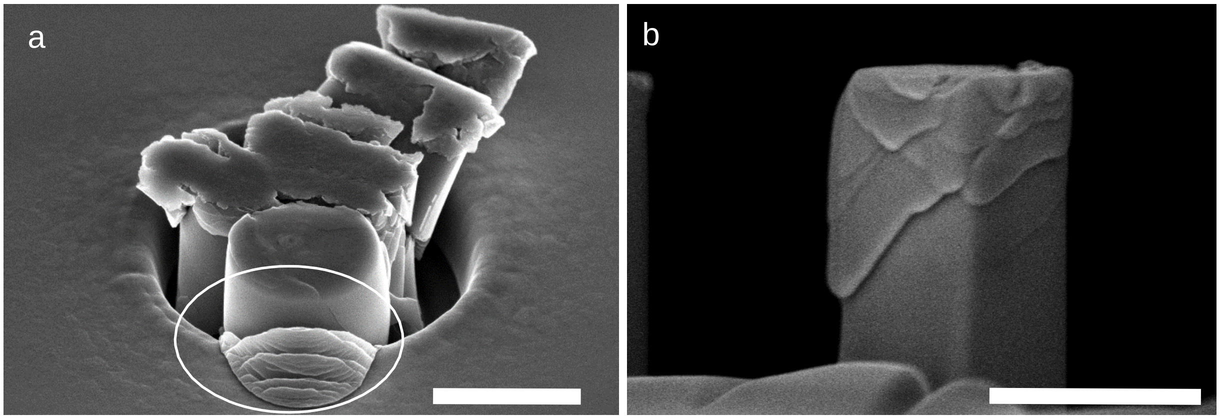

Supplementary Figure 3. Typical SEM images of the post-deformed pillars. Deformation bands with wavy morphology are apparent. Scale bars, a, 1 µm and, b, 500 nm.

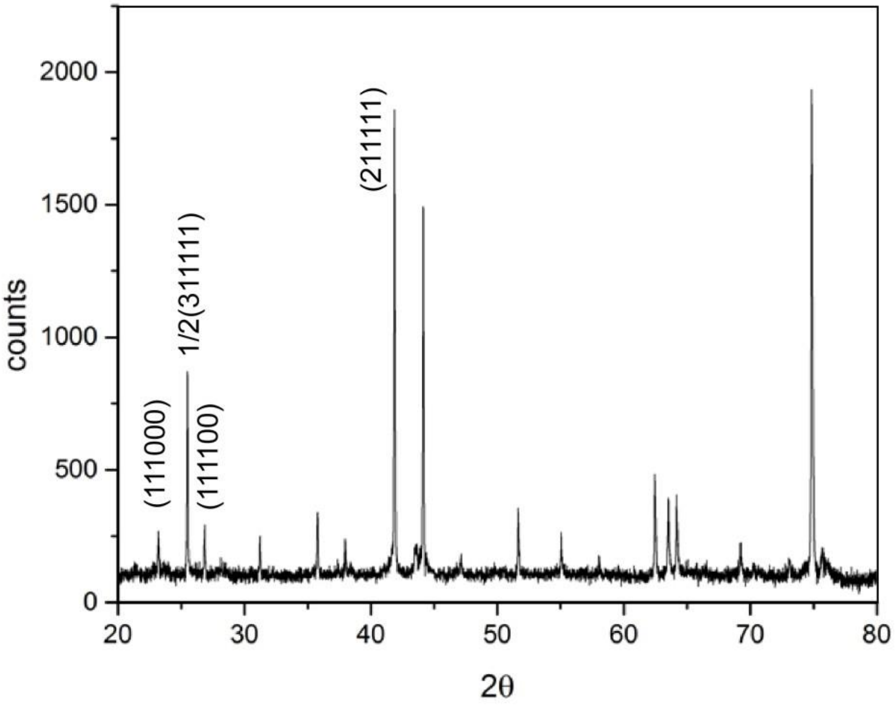

Supplementary Figure 4. **Powder diffraction pattern of as-cast i-Al-Pd-Mn.** Pattern, collected under Cu-$K_{\alpha1}$ beam, indicates an icosahedral phase and is comparable to a pattern from the literature[60].

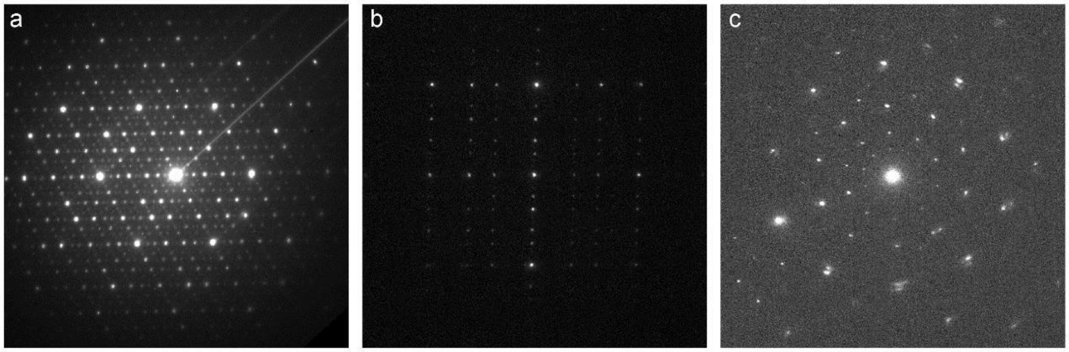

Supplementary Figure 5. **Electron diffraction patterns of the i-Al-Pd-Mn pillar.** Patterns along (a) threefold, (b) twofold and (c) fivefold symmetry axis, indicating that the as prepared pillar is a single icosahedral quasicrystal.

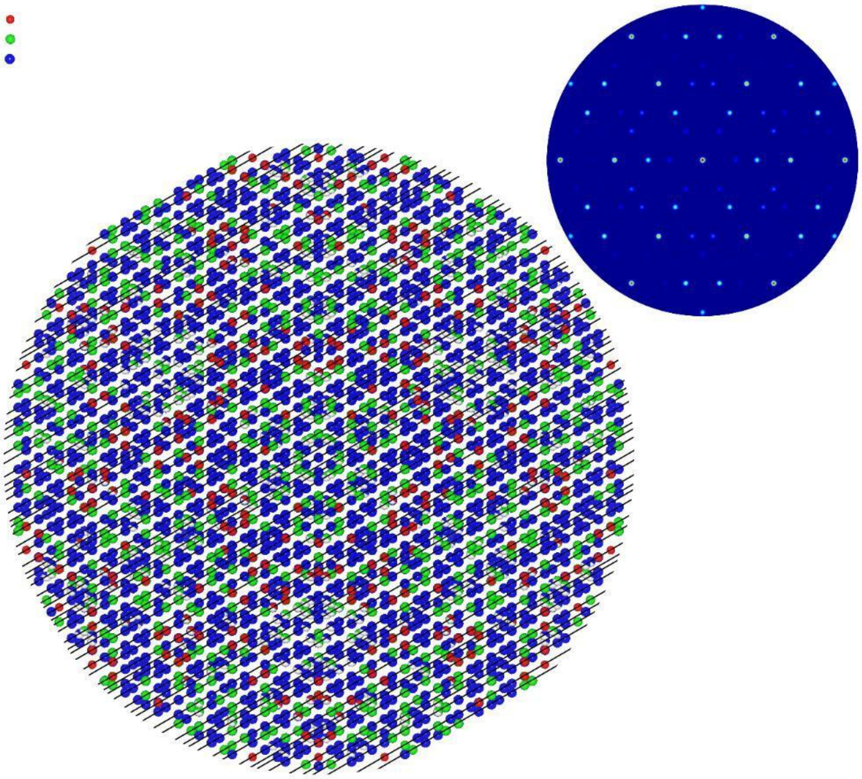

Supplementary Figure 6. **Quiquandon-Gratias atomic model of icosahedral Al-Pd-Mn.** Red circles represent Mn atoms, green–Pd, and blue–Al. The model is viewed along the threefold axis and produces a simulated pattern matching the observed electron diffraction pattern.
